# A Two-center Study on Facial Morphology in Patients With Complete Bilateral Cleft Lip, Alveolus, and Palate at the End of Growth: A Cross-sectional Cephalometric Study

**DOI:** 10.1097/SCS.0000000000011374

**Published:** 2025-04-18

**Authors:** Xander S. van Koot, Maria C. Meazzini, Marta Calò, Ewald M. Bronkhorst, Luca Autelitano, Edwin M. Ongkosuwito, Anne Marie Kuijpers-Jagtman, Mette A.R. Kuijpers

**Affiliations:** * Department of Orthodontics, University Medical Center Groningen, University of Groningen, Groningen, The Netherlands; † Department of Maxillo-Facial Surgery, Smile House, Regional Center for CLP, San Paolo e Carlo Hospital, University of Milano, Milano, Italy; ‡ Department of Dentistry, Radboud University Medical Center; § Department of Dentistry Section of Orthodontics and Craniofacial Biology; ∥ Cleft Palate Craniofacial Center, Radboudumc Amalia Children’s Hospital, Radboud University Medical Center, Nijmegen, The Netherlands; ¶ Department of Orthodontics and Dentofacial Orthopedics, School of Dental Medicine, University of Bern, Bern, Switzerland; # Faculty of Dentistry, Universitas Indonesia, Campus Salemba, Jakarta, Indonesia

**Keywords:** Cephalometry, cleft lip/surgery, cleft palate/surgery, craniofacial abnormalities, orthodontic treatment, orthodontics

## Abstract

**Objective:**

To compare the dentofacial morphology of patients with complete bilateral cleft lip, alveolus, and palate (CBCLAP) treated at 2 European centers participating in the ERN CRANIO Network, employing different protocols for alveolar cleft closure. The Milano Cleft Center (Center M) utilizes early secondary gingivo-periosteo-plasty (esGPP) at the time of hard palate closure, while the Nijmegen Cleft Center (Center N) employs early secondary alveolar bone grafting with or without premaxillary osteotomy at 9 to 11 years of age.

**Materials and Methods:**

This retrospective long-term outcome study included 95 patients (44 from Center M; 51 from Center N), treated consecutively from birth on in the same center, and evaluated at the end of growth (mean age 18 y). Lateral cephalograms were used to analyze skeletal and dentoalveolar variables. Measurements were conducted by an independent hospital to ensure objectivity. Statistical analyses included *t* tests and multiple linear regression to assess the effects of center, protocol, age, and sex.

**Results:**

The skeletal variable SN-ML (mandibular plane angle) indicated a more hyperdivergent pattern in Center N (mean 37.65 degrees, SD=6.62) compared with Center M (mean 34.55°, SD 7.52; *P*=0.037). Among dentoalveolar variables, only the upper incisor inclination (ILs-NL) was significantly larger in Center M (4.06 degrees, *P*=0.05). Regression analysis revealed minimal effects of center, protocol, age, or sex on skeletal and dentoalveolar variables.

**Conclusion:**

Patients from both centers achieved acceptable maxillofacial outcomes independent of the type of alveolar cleft closure. Further research should consider patient-reported outcomes to align clinical evaluations with patient perceptions.

Complete bilateral cleft lip, alveolus, and palate (CBCLAP) represents the most severe subtype of the common orofacial clefts. The treatment of the deformity necessitates a comprehensive interdisciplinary approach that begins at birth and extends into adolescence. There is a wide range of approaches to managing CBCLAP, each with its own impact on craniofacial development and long-term outcomes. Current protocols aim at optimizing the quality of treatment outcomes while at the same time reducing the burden of care. In general, patients are followed from birth to 18 years of age by a multidisciplinary cleft team composed of specialists in pediatrics, plastic and/or maxillofacial surgery, otolaryngology, speech and language therapy, clinical genetics, pediatric dentistry and prosthodontics, orthodontics, psychology, and social nursing. All the above members of the team have their own specific roles.^1,2^

From the orthodontic point of view, the primary challenge lies in managing the associated impairments in maxillofacial growth, which differ significantly between patients with or without a cleft.^3,4^ Newborns with CBCLAP may have a severely protruded premaxilla with distorted nasal anatomy. These babies may benefit from presurgical infant orthopedics with or without nasoalveolar molding (NAM), but the effects of this therapy in CBCLAP are still debated.^5,6^ After eventual infant orthopedics, primary lip repair and palate repair are performed. Between the ages of 6 and 9 years, maxillary expansion may be performed before early secondary alveolar bone grafting (SABG). This procedure can be combined with an osteotomy of the premaxilla and/or fistula closure or closure of the hard palate.^7,8^ An alternative to SABG is a gingivoperiosteoplasty (GPP), typically performed either as a primary procedure at 6 months during lip closure or at 2.5-3 years of age during hard palate closure.^9^ This procedure promotes bone formation in the alveolar cleft by surgical repositioning of the muco-periosteal edges, potentially eliminating the need for bone grafting.^10,11^ The final orthodontic treatment starts during the mixed dentition period and may extend into late adolescence if jaw surgery is needed.^2,12^

There is considerable variability in the management of orofacial clefts.^13^ The burden of care can be very different between different treatment protocols. Given the low incidence of CBCLAP,^14^ comparing the treatment outcome of various treatment protocols employed across different cleft centers globally is crucial. For example, research networks like the European Reference Network ERN-CRANIO,^15^ Americleft^16^ and Scandcleft,^17^ pool together knowledge and expertize and promote intercenter comparisons. In the current study, we focus on the treatment outcomes regarding maxillofacial growth in CBCLAP in 2 European centers with fundamentally different approaches to the surgical closure of the alveolar clefts. The Milano Cleft Center and the Nijmegen Cleft Center have both developed distinct protocols and employed these for many years. The motivation for this study arose from the observation that patients with a history of CBCLAP from these European Cleft Centers appeared to achieve acceptable clinical outcomes in young adulthood, despite being treated under entirely different protocols. In the Milano Cleft Center, a 2-step protocol including primary cheilorhinoplasty and soft palate repair at 6 months and a second surgical step including early secondary gingivoperiosteoplasty (esGPP) and simultaneous hard palate closure at ~2.5 to 3 years of age is used. This approach has shown promising results with adequate ossification in both the alveolar and the nasal regions and normal rates of permanent tooth eruption.^18^ In unilateral cleft lip and palate (UCLP) only 5% of the patients treated according to this protocol have required a secondary alveolar bone graft.^11^ Conversely, the Nijmegen Cleft Center has a more classic approach for alveolar reconstruction, involving as a third surgical step, secondary alveolar bone grafting (SABG) of both clefts before eruption of the canines, mostly combined with an osteotomy of the premaxilla to ensure proper position of the premaxilla in the maxillary arch at the age of 9 to 11 years.^19^ Several long-term follow-up studies have shown that craniofacial growth outcomes in Nijmegen were comparable to those of Scandinavian cleft centers (Oslo and Gothenborg), which employ different treatment protocols.^7,20–22^

This study aims to compare the craniofacial morphology of patients with a complete bilateral cleft lip, alveolus, and palate (CBCLAP) at the end of the adolescent growth period who were consecutively treated in these 2 European cleft centers with different protocols for alveolar cleft closure.

## PATIENTS AND METHODS

### Ethical Approval

This retrospective cohort study involved a 2-center outcome analysis with outcome evaluation by an independent academic hospital. Two cleft centers participated in this study: (1) Smile House, Regional Center for CLP, San Paolo e Carlo Hospital, Milano, Italy (center M); and (2) Cleft Palate Craniofacial Center, Amalia Children’s Hospital, Radboud University medical center, Nijmegen, The Netherlands (center N). This research was conducted in accordance with the second Helsinki Declaration regarding research in human subjects. The Medical Ethical Committee East-Netherlands (METC Oost-Nederland) declared this study is not clinical research with human subjects as meant in the Medical Research Involving Human Subjects Act (WMO) (number 2024-17210, 09-04-2024).

### Subjects

Lateral cephalograms were available for 95 patients with CBCLAP consecutively treated from birth in the same center by the same surgeons. From center M, 44 patients were included, born between 1989 and 2005. From center N, 51 patients were included, born between 1987 and 2004. Cephalograms were evaluated at the age of ~18 years. The age of 18 was chosen because it is before an eventual maxillary osteotomy, and facial growth has ceased in most patients.^23^

Inclusion criteria were CBCLAP with a confirmed diagnosis of CBCLAP through preoperative (written) records and neonatal extra-oral pictures and/or maxillary dental casts; presence of Simonart’s bands allowed if hard tissue union was not present; Caucasian ethnicity; no congenital malformations, no syndromes; and treated from birth onwards in the same center by the same experienced surgeons.

### Treatment Protocol

Supplemental Table 1, Supplemental Digital Content 1, http://links.lww.com/SCS/H714 shows the treatment protocols, regarding the surgical and orthodontic procedures for CBCLAP, of the 2 centers.

In center M, from 1989 to 1998, patients with CBCLAP received infant orthopedics with a passive plate and lip taping. Lip repair was performed with a modified Delaire cheiloplasty and soft palate repair (modified Pigott’s). No primary rhinoplasty was carried out. The hard palate was repaired together with an esGPP.^9^ A secondary columella elongation was performed in most patients during early childhood. From 1999, all patients received a modified Figueroa’s type nasoalveolar molding (NAM),^24^ which aims at nasal molding and does not require close approximation of the alveolar segments. Patients were seen at monthly visits. At ~6 months of age, lip surgery (modified Delaire’s) and a Cutting primary rhinoplasty^25^ were carried out together with soft palate closure (modified Pigott’s). The hard palate was repaired together with esGPP (early secondary GPP) at ~3.5 years of age. All children received comprehensive orthodontic treatment and at the end of adolescence growth orthodontic and surgical management (osteotomy, scar revision and/or rhinoplasty) if necessary.

Treatment in center N started with infant orthopedics, consisting of a passive plate with extra-oral strapping.^7^ At 6 to 7 months of age, a 1-stage lip closure surgery (modified Manchester) was performed. At 12 to 18 months of age, the soft palate was reconstructed using the modified Von Langenbeck technique. Between the ages of 6 and 9, early orthodontic interventions took place if necessary (including Hyrax and facemask). Around the age of 9 to 11 years, before eruption of the canines, alveolar bone grafting of both clefts was performed together with hard palate closure and combined with an osteotomy of the premaxilla, if needed, to ensure proper position of the premaxilla in the maxillary arch.^19^ After surgery was performed, comprehensive orthodontic treatment was done in all children. At the end of growth orthodontic and surgical management took place (osteotomy, scar revision, and/or rhinoplasty) if necessary.

### Radiographic Cephalometric Assessment

**FIGURE 1:**
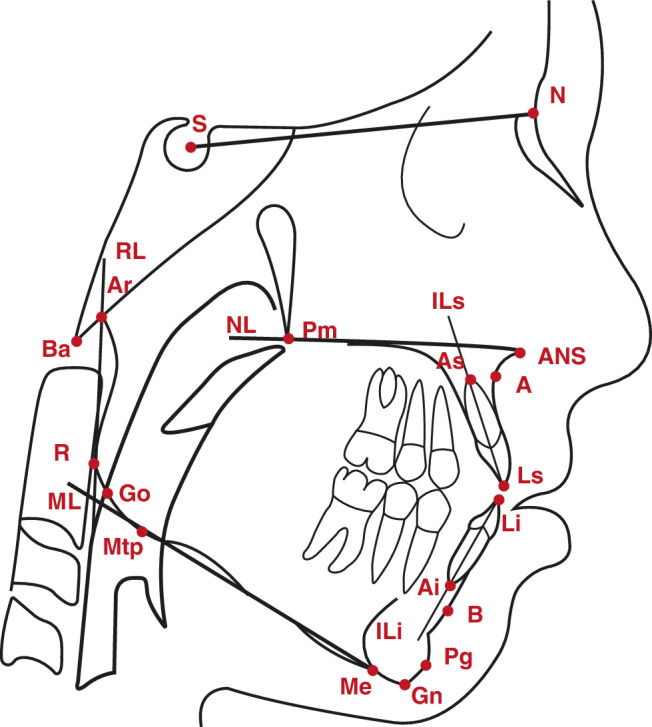
Cephalometric reference points on the lateral cephalogram (adapted from Bartzela et al 2012).^7^

Lateral cephalograms were taken in centric occlusion and oriented with the Frankfurt horizontal plane parallel to the floor. The cephalometric measurements were conducted by 1 experienced observer (X.v.K.) from a third hospital who was not involved in the patients’ treatment. A commercially available software program for cephalometric analysis was used (Viewbox 4, Dhal software, Kifissia, Greece). A total of 18 cephalometric reference points were identified (Fig. 1, Supplemental Table 2, Supplemental Digital Content 2, http://links.lww.com/SCS/H715). From these reference points, 6 reference lines were defined, and skeletal sagittal (n=4), skeletal vertical (n=5), and dentoalveolar (n=4) variables were calculated (Supplemental Table 3, Supplemental Digital Content 3, http://links.lww.com/SCS/H716). To avoid errors due to magnification differences between the 2 treatment centers, only angular measurements were used.

To determine intraobserver reliability, 10 cephalograms were randomly selected from each center and digitized twice by the same observer after a 4-week interval.

### Statistical Analysis

The statistical analysis was performed using SPSS version 29.0.2.0 for Windows (IBM). To assess intraobserver reliability, the reliability coefficients between the 2 measurements of the observer were calculated as Pearson’s correlation coefficients. Paired Sample *t* tests were applied to identify systematic differences between the first and the second measurement. The duplicate measurement error (DME) was calculated as the SD of the difference between 2 observations divided by √2. In addition, Bland–Altman plots were generated for each variable.

Means and differences between means, SD, and 95% confidence intervals were calculated for all variables. Differences between centers were tested using the *t* test for independent samples. The difference in the rate of orthognathic surgery between centers was tested with the 2-proportion z-test. The level of significance was set at *P*≤0.05.

In the multiple linear regression analysis, the effects of center, age at evaluation, and sex (independent variables) on the cephalometric variables were explored. As the treatment protocol in Milano changed from birth year 1999 on, birth year ≥1999 in center M was also included in the regression analysis. The adjusted *R* ^2^ values were used to interpret the outcomes.

## RESULTS

### Sample

A total of 95 patients were included in this study; 44 patients were treated in Center M and 51 patients in Center N. The sample characteristics are shown in Supplemental Table 4, Supplemental Digital Content 4, http://links.lww.com/SCS/H717. Of the 44 patients in Center M, 29 (65.9%) were male. In Center N there were 38 males (74.5%). The mean age at evaluation in Center M was 19.1 years (SD 3.6) and in Center N 18.4 years (SD 2.8). In Center M, 2 operations were performed to close the lip, palate, and alveolar clefts, while in Center N, 3 operations were conducted to achieve this goal (Supplemental Tables 1, Supplemental Digital Content 1, http://links.lww.com/SCS/H714 and 4, Supplemental Digital Content 4, http://links.lww.com/SCS/H717). Orthognathic surgery in center M was suggested in 19.5% of the sample, and in center N in 37.3%. The test statistic (z) was −1.91 (*P*=0.057). This means there is not enough statistical evidence to conclude that the proportions of patients suggested for orthognathic surgery in centers M and N are significantly different.

### Error of the Method

**FIGURE 2:**
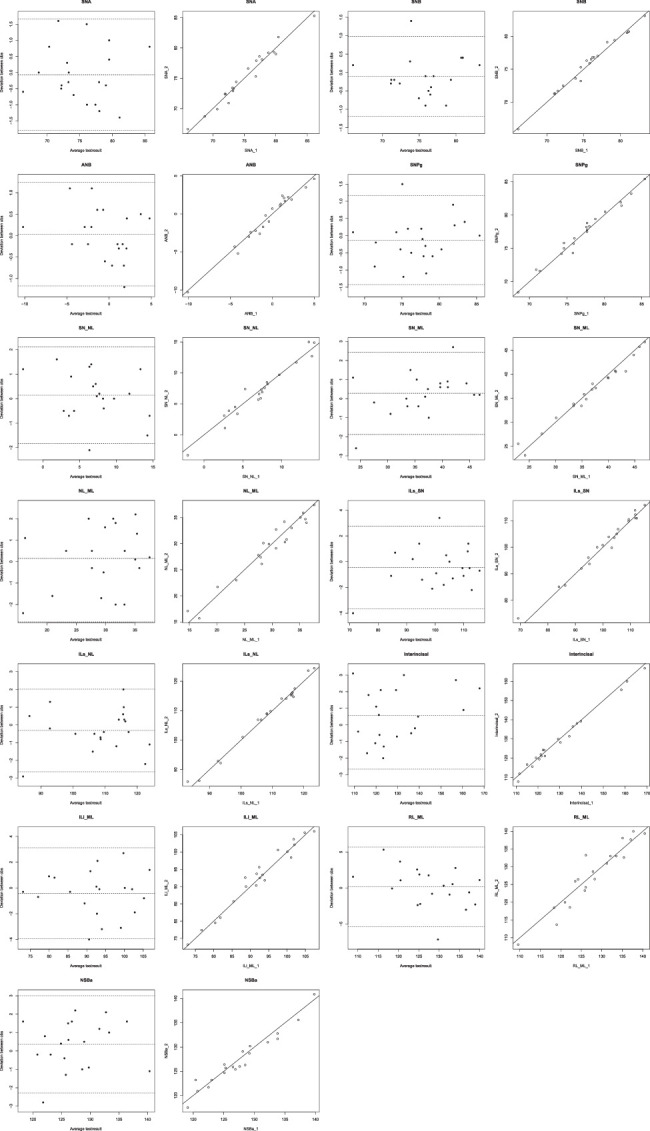
Bland-Altman and scatter plots intraobserver.

The intraobserver reliability and the measurement errors are shown in Supplemental Table 5, Supplemental Digital Content 5, http://links.lww.com/SCS/H718. The reliability coefficients ranged from 0.95 to 0.99. The largest variability (DME) was found for angle RL-ML (1.96 degrees). There were no significant differences (*P*<0.05) between the first and the second measurements for any of the variables (Fig. 2). Bland–Altman plots showed that most differences between measurements were within the mean ±1.96 SD with only a few outliers. No systematic bias was observed (Supplemental Table 5, Supplemental Digital Content 5, http://links.lww.com/SCS/H718).

### Cephalometric Outcomes

The results for the cephalometric variables are shown in Supplemental Table 6, Supplemental Digital Content 6, http://links.lww.com/SCS/H719. For the skeletal variables, only SN-ML differed significantly between the centers (mean difference −3.10 degrees; *P*=0.037). The mean SN-ML angle in Center M was 34.55 degrees (SD=7.52), while in Center N, it was 37.65 degrees (SD=6.62), pointing to a hyperdivergent skeletal pattern in the latter center. In the category “dentoalveolar variables,” ILs-NL was 4.60 degrees larger in Center M than in Center N, a difference that just reached significance (*P*=0.05).

The results of the multiple regression model are shown in Supplemental Table 7, Supplemental Digital Content 7, http://links.lww.com/SCS/H720. The analysis showed a few significant sex effects in the skeletal variables (SNA and ANB angle), but the explained variance was very low. There were no significant effects of the independent variables center, age, or sex on the dentoalveolar variables. However, protocol in Center M from 1999 onwards had an effect on outcome for 1 skeletal sagittal cephalometric variables (SNA), 1 skeletal vertical (NaSBa), and 2 dentoalveolar variables (Ils-SN, Ils-NL), but all explained variances were very low. The highest explained variance was found for the angle Ils-NL (adjusted *R* ^2^=0.067).

## DISCUSSION

This study aimed to compare the craniofacial morphology of patients with a complete bilateral cleft lip, alveolus, and palate (CBCLAP) at the end of the growth period, consecutively treated in 2 European cleft centers with different protocols regarding the closure of the alveolar clefts. Despite the different treatment approaches, there were only minor differences in craniofacial morphology between the 2 centers when the patients were grown up. The proportions of orthognathic surgery at the end of the growth period were also not significantly different between centers M and N (20% and 37%, respectively). Skeletally, patients only differed for the mandibular plane angle (SN-ML), which was 3.10 degrees larger in Center N, pointing to a hyperdivergent growth pattern in the Dutch sample. The difference may be related to inherent population differences between the 2 centers. Earlier studies have shown cephalometric differences between populations, reflecting racial and genetic differences.^26,27^ The significant difference in the IIs-NL angle (4.60 degrees less in Nijmegen, *P*=0.050) could also be explained by dental compensation for the relative more Class II facial configuration in the patients in Northern Europe or more dental compensation for Class III facial configuration in center M.

As mentioned, the differences in protocols did not lead to significant skeletal differences at the end of facial growth. Even though an often-named concern of GPP is iatrogenic facial growth restriction through early scar formation and possibly subsequent malocclusion due to early closure and scarring of the alveolar arch. Such restriction could increase the need for orthodontic and orthognathic treatment at the time of skeletal maturity.^28^ Henkel and Gundlach^29^ reported severe maxillary retrusion in a large sample of 146 patients treated with primary GPP and followed till 16 years of age. Similar detrimental effects of primary GPP were reported by Hsieh et al.^30^ Long-term results in terms of facial growth after primary GPP (performed at the age of 6 mo with cheilorhinoplasty) showed a much higher need for orthognathic surgery than in our study, which was up to 83.3%.^31^ Even taking into consideration that parameters for indication for orthognathic surgery are not standardized and might be different between centers, there is a doubtless significant impact of the early GPP protocol on growth. Therefore, several centers prefer to allow for a longer period of undisturbed growth of the maxilla before performing alveolar bone grafting, such as in the current Milano protocol where the esGPP is combined with hard palate closure at a later age. However, it was shown that patients with a complete unilateral cleft lip, alveolus, and palate (CUCLAP) undergoing esGPP at 2 years of age have a higher need for final orthognatic surgery in comparison with the Oslo surgical protocol with a 13% need for osteotomies in the Oslo sample and a 26% need in the Milano esGPP sample.^32^

Besides different protocols for closure of the alveolar clefts, there is also a difference in the timing of palatal closure between the 2 centers, which should be taken into consideration. In the Nijmegen protocol, the 2-stage palatal closure was completed at ~11 years of age when also SBAG was performed, while in the Milano center, hard palate closure was performed together with esGGP at ~3 ½ years of age. Nevertheless, the skeletal growth seems similar between the 2 centers. This might suggest that late closure of the hard palate resulting in palatal scar tissue shortly before the pubertal growth spurt may have a similar effect on maxillary growth as closing early at ~3 ½ years of age. The findings of the Scandcleft randomized controlled trials into the effect of different timings of palatal surgery in CUCLAP also demonstrated that timing was of minor importance for facial growth.^33^ However, the size of the remaining cleft in the hard palate did affect maxillary growth, and therefore, it is recommended that the timing of closure should be based on the hard palate cleft size.^34^ This is in accordance with Berkowitz’s studies on palatal growth that concluded that the timing of cleft palate closure should be based on the ratio of the area of the cleft to that of the palatal segments and that palatal growth velocity starts slowing down only after 27 to 30 months.^35^ Therefore, delaying GPP after 30 months of age might explain the lower impact of esGPP compared with that of primary GPP at 6 months.

GPP is associated with a reduced number of surgeries to close the alveolar cleft. The earliest data became available through work of Millard who later reported a non-documented need for bone grafting of only 3%.^36^ Santiago et al^37^ reported benefits of primary GPP with reduction up to 60% of secondary alveolar bone grafting (SABG) at the time of the mixed dentition in the primary GPP group. Matic et al^38^ reported a success rate of primary GPP of 40%, comparing it to SABG which had a success rate of 88%. GPP performed at a later stage seems to have a higher success rate. This was suggested by Delaire, who first introduced early secondary GPP performed at a later time with hard palate closure but never reported actual data on ossification.^39^ Meazzini et al^11,18^ reported on the results of early secondary GPP performed at the time of hard palate closure, at 2.5 years of age in CUCLAP and at 3.5 years in patients with CBCLAP. Their ossification studies show an extremely low need for secondary bone grafting. In the present study, in the Milano sample, 3 out of 44 patients (6%) needed ABG, 2 on 1 side, and only 1 on both sides, in total 4 sides out of 88 clefts (4.5% needed for SABG). The success rate for early secondary ABG in the Nijmegen sample was 93.3% and no repeat procedures were needed. In 7 out of the 104 clefts (6.7%), the bone height at 1 side was insufficient to support tooth movement into the grafted area or to place a dental implant. These cases were managed with an adhesive bridge, except for 1 patient who required a frame prosthesis due to multiple missing teeth. This success rate aligns with a previous Nijmegen study, which reported a 91% success rate for early secondary ABG, primarily using rib grafts.^40^

The findings of the current study in CBCLAP demonstrated that both treatment protocols allow for similar maxillary growth, however, with a different burden of surgical care, given that lip, nose, palate, and alveolus are repaired in 2 steps (Milano) instead of 3 (Nijmegen). Further studies will need to assess results for the soft tissues. Also, the burden of orthodontic care requires further study. However, burden of care studies are difficult to perform^41^ because there are many variables involved and many views regarding additional procedures, such as NAM and orthodontics in general. However, attempts must be made to collect additional data that could support findings of current research or could expand our views on current treatment protocols.

### Limitations

This study has some limitations. Firstly, it is a retrospective study that has inherent disadvantages like selection bias and unmeasured confounding variables.^42^ In our study, we attempted to minimize biases by focusing on consecutively treated cases managed by the same CLP team. This approach ensures some level of consistency in treatment protocols and data recording practices, improving the reliability of the findings despite the inherent limitations. Furthermore, as mentioned earlier, the incidence of CBCLAP is low, and because of prenatal screening, it is decreasing. For example, in the Netherlands, the number of patients with CBCLAP decreased from N=47 registered cases in 2012 to N=12 in 2021.^43^ This makes it impossible to perform a randomized controlled trial or a prospective comparative longitudinal cohort study until growth has ceased.

Secondly, we used 2D cephalometry in this study. Three-dimensional cephalometry represents the most advanced method for analyzing facial structures; however, due to concerns regarding radiation exposure associated with traditional multislice or cone-beam computed tomography (CBCT), 2D cephalometry is likely to remain a key tool for conducting studies for evaluating facial morphology and growth in individuals with cleft lip and palate. However, advancements in ultra-low-dose CBCT technology present a promising future alternative, as it offers significantly reduced radiation exposure while still providing comprehensive 3-dimensional imaging.^44^

Lastly, in this study, we examined the potential differences in the burden of care associated with 2 treatment protocols, focusing specifically on data obtained from cephalometric measurements. However, perspectives or opinions of patients or parents were not explored. This raises the possibility that what we consider “marginal and clinically unimportant” may differ significantly from patients’ perceptions. Future research should prioritize gathering patient opinions on clinical outcomes to better understand their perspectives.

## CONCLUSIONS

Patients from both centers achieved acceptable maxillofacial growth outcomes. Center M seems to obtain the same skeletal result with a lower primary surgical burden, but other aspects, such as orthodontic or speech burden of care, will need to be taken into consideration. Further research should consider patient-reported outcomes to align clinical evaluations with patient perceptions.

## Supplementary Material

SUPPLEMENTARY MATERIAL
