# Supplementary material for: A Two-center Study on Facial Morphology in Patients With Complete Bilateral Cleft Lip, Alveolus, and Palate at the End of Growth: A Cross-sectional Cephalometric Study
Source: J Craniofac Surg. 2025 Apr 18;36(8):2938–43. doi: 10.1097/SCS.0000000000011374 (PMC12537043; doi:10.1097/SCS.0000000000011374)
Supplement: SUPPLEMENTARY MATERIAL [file scs-36-02938-s001.docx]

**Supplemental Table 1** Treatment protocols (surgery and orthodontics) for patients with a complete bilateral cleft lip, alveolus and palate from birth until 20 years of age of the cleft palate centers in this study.

| Planned age | Center M | Center N |
| --- | --- | --- |
| Birth | (before 1999) Infant orthopedics with passive plate and lip taping.  (from 1999) Nasoalveolar molding (NAM Figueroa’s type, passive plate with nasal supports and strapping) | Infant orthopedics with passive plate and extra-oral strapping |
| 6-7 months | (before 1999) Lip repair with a modified Delaire cheiloplasty and soft palate repair (modified Pigott’s).  (from 1999) Lip repair with a modified Delaire cheiloplasty and a Cutting primary rhinoplasty and soft palate repair (Sommerlad) | One-stage lip closure (modified Manchester) |
| 12-18 months |  | Modified Von Langenbeck soft palate closure |
| 18 – 42 months | (before 1999) Hard palate repair with early secondary gingivo-periosteoplasty (esGPP) and columella elongation  (from 1999) Hard palate repair with early secondary gingivoperiosteo-plasty at 42 months (esGPP) |  |
| 6-9 years |  | Early orthodontic interventions (e.g. Hyrax and/or facemask) |
| 8-13 years |  | Hard palate closure and bilateral alveolar bone grafting and osteotomy of the premaxilla (if needed) |
| 12-15 years | Comprehensive orthodontic interventions (e.g. fixed appliances) | Comprehensive orthodontic interventions (e.g. fixed appliances) |
| 17-20 years | Orthodontic and surgical management (e.g. osteotomy, scar revision and/or rhinoplasty) | Orthodontic and surgical management (e.g. osteotomy, scar revision and/or rhinoplasty) |

Center M= Milano; Center N=Nijmegen
