# Supplementary material for: A Two-center Study on Facial Morphology in Patients With Complete Bilateral Cleft Lip, Alveolus, and Palate at the End of Growth: A Cross-sectional Cephalometric Study
Source: J Craniofac Surg. 2025 Apr 18;36(8):2938–43. doi: 10.1097/SCS.0000000000011374 (PMC12537043; doi:10.1097/SCS.0000000000011374)
Supplement: SUPPLEMENTARY MATERIAL [file scs-36-02938-s002.docx]

**Supplemental Table 2** Cephalometric reference points and lines

| **Point** | **Name** | **Reference points / lines** |  |
| --- | --- | --- | --- |
| **Skeletal and dental** | | | |
| S | Sella | Geometric centre of the Sella turcica |  |
| N | Nasion | Most anterior point at the frontonasial suture |  |
| ANS | Point ANS | Anterior Nasal Spine |  |
| A | Point A | Deepest point on the anterior contour of the upper alveolar process |  |
| As | Apex superius | Apex of the root of the upper central incisor |  |
| Ls | Incision superius | Incisal edge of the most prominent upper incisor |  |
| Li | Incision inferius | Incisal edge of the most prominent lower incisor |  |
| Ai | Apex inferius | Apex of the root of lower central incisor |  |
| B | Point B | Deepest point of the anterior contour of the lower alveolar process |  |
| Pg | Pogonion | Most anterior point of the mandibular symphysis |  |
| Gn | Gnathion | Most anterior inferior point of the bony chin |  |
| Me | Menton | Most inferior point of the mandibular symphysis |  |
| Go | Gonion | Most posterior inferior point on the angle of the mandible. |  |
| Mtp | Mandibular Tangent Posterior | Most posterior inferior point on the outline of the mandibular body |  |
| R | Ramus point | Most posterior-inferior point of the mandibular ramus |  |
| Ar | Articulare | Constructed point at the intersection of the images of the posterior margin of the ramus and the outer margin of the cranial base |  |
| Ba | Basion | Lowest point on the anterior margin of the foramen magnum in the median plane |  |
| Pm | Pterygo-maxillare | Intersection of the nasal floor and the apex of the pterygomaxillary fissure |  |
| **Reference lines** | | | |
| SN | Sella-Nasion | Sella-Nasion line |  |
| NL | Nasal Line | Line through Pm and ANS |  |
| ILs | Axis upper incisors | Line through incisal edge and apex of the root |  |
| ILi | Axis lower incisors | Line through incisal edge and apex of the root |  |
| ML | Mandibular Line | Tangent of the lower border of the mandible through Me and Mtp |  |
| RL | Ramus Line | Line through Ar and R |  |
