# Supplementary material for: A Two-center Study on Facial Morphology in Patients With Complete Bilateral Cleft Lip, Alveolus, and Palate at the End of Growth: A Cross-sectional Cephalometric Study
Source: J Craniofac Surg. 2025 Apr 18;36(8):2938–43. doi: 10.1097/SCS.0000000000011374 (PMC12537043; doi:10.1097/SCS.0000000000011374)
Supplement: SUPPLEMENTARY MATERIAL [file scs-36-02938-s003.docx]

**Supplemental Table 3** Cephalometric variables (angles) derived from reference points and lines (see Figure 1) and supplementary Table 1

| **Cephalometric Variables** |
| --- |
| **Skeletal sagittal** |
| SNA |
| SNB |
| ANB |
| SNPg |
| **Skeletal vertical** |
| SN-NL |
| SN-ML |
| NL-ML |
| RL-ML |
| NSBa |
| **Dentoalveolar** |
| ILs-SN |
| ILs-NL |
| Interincisal |
| ILi-ML |
