# Supplementary material for: A Two-center Study on Facial Morphology in Patients With Complete Bilateral Cleft Lip, Alveolus, and Palate at the End of Growth: A Cross-sectional Cephalometric Study
Source: J Craniofac Surg. 2025 Apr 18;36(8):2938–43. doi: 10.1097/SCS.0000000000011374 (PMC12537043; doi:10.1097/SCS.0000000000011374)
Supplement: SUPPLEMENTARY MATERIAL [file scs-36-02938-s004.docx]

| **Supplemental Table 4** Sample characteristics |  |  |
| --- | --- | --- |
|  | Center M | Center N |
| Sample |  |  |
| Total n | 44 | 51 |
| Patients born before 1999 | 17 |  |
| Patients born in or after 1999 | 27 |  |
| Simonart's band, two-sided/one-sided | 5/7 | 2/3 |
| Male/Female | 29/15 | 38/13 |
| Mean age ± SD (yr) | 19.1 ± 3.6 | 18.4 ± 2.8 |
| Operations (mean age ± SD in yr) |  |  |
| Primary cheilo-rhinoplasty + primary columella elongation  + soft palate closure | 0.7 ± 0.3 |  |
| Primary lip closure |  | 0.7 ± 1.3 |
| Soft palate closure |  | 1.5 ± 1.9 |
| Hard palate closure + esGAP | 3.7 ± 1.7 |  |
| Hard palate closure + bone grafting (n=14) |  | 11.0 ± 3.3 |
| Hard palate closure + bone grafting + osteotomy of premaxilla (n=37) | | 10.6 ± 1.8 |
| M= Milano; N=Nijmegen |  |  |
