# Supplementary material for: A Two-center Study on Facial Morphology in Patients With Complete Bilateral Cleft Lip, Alveolus, and Palate at the End of Growth: A Cross-sectional Cephalometric Study
Source: J Craniofac Surg. 2025 Apr 18;36(8):2938–43. doi: 10.1097/SCS.0000000000011374 (PMC12537043; doi:10.1097/SCS.0000000000011374)
Supplement: SUPPLEMENTARY MATERIAL [file scs-36-02938-s005.docx]

| **Supplemental Table 5** Intra-observer reliability for hard tissue cephalometric measurements (degrees) | | | | | |  |
| --- | --- | --- | --- | --- | --- | --- |
|  | Reliability | DME | Mean difference | 95% CI | *P-*value |  |
| **Skeletal sagital** | |  |  |  |  |  |
| SNA | 0.98 | 0.61 | -0.07 | [-0.48...0.34] | 0.722 |  |
| SNB | 0.99 | 0.38 | -0.11 | [-0.36...0.15] | 0.397 |  |
| ANB | 0.99 | 0.43 | 0.04 | [-0.25...0.32] | 0.798 |  |
| SNPg | 0.99 | 0.46 | -0.13 | [-0.43...0.17] | 0.380 |  |
| **Skeletal vertical** | |  |  |  |  |  |
| SN-NL | 0.98 | 0.70 | 0.14 | [-0.32...0.60] | 0.533 |  |
| SN-ML | 0.99 | 0.76 | 0.28 | [-0.23...0.79] | 0.260 |  |
| NL-ML | 0.97 | 1.05 | 0.15 | [-0.54...0.84] | 0.655 |  |
| RL-ML | 0.95 | 1.96 | 0.15 | [-1.15...1.45] | 0.814 |  |
| NSBa | 0.97 | 0.94 | 0.36 | [-0.26...0.98] | 0.238 |  |
| **Dentoalveolar** | |  |  |  |  |  |
| ILs-SN | 0.99 | 1.14 | -0.46 | [-1.21...0.29] | 0.216 |  |
| ILs-NL | 0.99 | 0.82 | -0.31 | [-0.86...0.24] | 0.248 |  |
| Interincisal | 0.99 | 1.15 | 0.58 | [-0.18...1.34] | 0.126 |  |
| ILi-ML | 0.98 | 1.25 | -0.42 | [-1.25...0.41] | 0.302 |  |
| *DME* duplicate measurement error; *CI* confidence interval | | | | |  |  |
