# Supplementary material for: A Two-center Study on Facial Morphology in Patients With Complete Bilateral Cleft Lip, Alveolus, and Palate at the End of Growth: A Cross-sectional Cephalometric Study
Source: J Craniofac Surg. 2025 Apr 18;36(8):2938–43. doi: 10.1097/SCS.0000000000011374 (PMC12537043; doi:10.1097/SCS.0000000000011374)
Supplement: SUPPLEMENTARY MATERIAL [file scs-36-02938-s006.docx]

| **Supplemental Table 6** Differences between centers for hard tissue cephalometric variables (t-tests) | | | | | | |  |
| --- | --- | --- | --- | --- | --- | --- | --- |
|  | Center M Mean (SD) | Center N Mean (SD) | Difference | 95% CI  lower limit | 95% CI  upper limit | *P-*value |  |
| **Skeletal sagital** | |  |  |  |  |  |  |
| SNA | 75.24 (5.90) | 74.93 (4.46) | 0.31 | -1.85 | 2.47 | 0.775 |  |
| SNB | 76.20 (4.59) | 75.23 (5.26) | 0.97 | -1.04 | 2.98 | 0.339 |  |
| ANB | -0.97 (3.35) | -0.31 (3.31) | -0.66 | -2.02 | 0.70 | 0.341 |  |
| SNPg | 77.83 (4.52) | 76.38 (5.43) | 1.45 | -0.58 | 3.48 | 0.159 |  |
| **Skeletal vertical** | |  |  |  |  |  |  |
| SN-NL | 7.75 (3.97) | 7.35 (4.63) | 0.39 | -1.36 | 2.15 | 0.657 |  |
| SN-ML | 34.55 (7.52) | 37.65 (6.62) | -3.10 | -6.01 | -0.19 | **0.037** |  |
| NL-ML | 27.49 (10.69) | 30.42 (6.92) | -2.93 | -6.68 | 0.82 | 0.124 |  |
| RL-ML | 127.50 (8.06) | 128.36 (7.80) | -0.86 | -4.11 | 2.38 | 0.598 |  |
| NSBa | 128.24 (6.01) | 128.26 (5.09) | -0.02 | -2.31 | 2.28 | 0.99 |  |
| **Dentoalveolar** | |  |  |  |  |  |  |
| ILs-SN | 105.74 (9.77) | 101.69 (12.13) | 4.06 | -0.41 | 8.52 | 0.075 |  |
| ILs-NL | 113.51 (10.30) | 108.92 (12.30) | 4.60 | -0.01 | 9.20 | **0.05** |  |
| Interincisal | 127.52 (12.87) | 130.46 (15.71) | -2.94 | -8.76 | 2.89 | 0.319 |  |
| ILi-ML | 92.17 (9.40) | 90.21 (7.61) | 1.96 | -1.57 | 5.49 | 0.272 |  |
| M=Milano; N=Nijmegen | |  |  |  |  |  |  |
