# Supplementary material for: A Two-center Study on Facial Morphology in Patients With Complete Bilateral Cleft Lip, Alveolus, and Palate at the End of Growth: A Cross-sectional Cephalometric Study
Source: J Craniofac Surg. 2025 Apr 18;36(8):2938–43. doi: 10.1097/SCS.0000000000011374 (PMC12537043; doi:10.1097/SCS.0000000000011374)
Supplement: SUPPLEMENTARY MATERIAL [file scs-36-02938-s007.docx]

| **Supplemental Table 7** Linear regression analysis to analyze the effects of center, Milano birth year, age, and sex on outcome variables | | | | | | |  |  |
| --- | --- | --- | --- | --- | --- | --- | --- | --- |
|  | Effect | Estimate | 95% CI lower limit | 95% CI upper limit | *P*-value | R^2^ |  |  |
| **Skeletal sagital** | | | | | | |  |  |
| SNA |  | 70.03 | 62.81 | 77.26 | 0.000 | 0.067 |  |  |
|  | Center M vs N | -2.11 | -4.97 | 0.74 | 0.145 |  |  |  |
|  | Milano born from 1999 onwards | 3.26 | 0.13 | 6.40 | **0.042** |  |  |  |
|  | Age | 0.26 | -0.07 | 0.58 | 0.123 |  |  |  |
|  | Sex M vs F | -2.60 | -4.83 | -0.37 | **0.023** |  |  |  |
| SNB |  | 72.24 | 65.06 | 79.42 | 0.000 | 0.005 |  |  |
|  | Center M vs N | -0.80 | -3.64 | 2.04 | 0.578 |  |  |  |
|  | Milano born from 1999 onwards | 2.56 | -0.55 | 5.68 | 0.105 |  |  |  |
|  | Age | 0.16 | -0.17 | 0.48 | 0.346 |  |  |  |
|  | Sex M vs F | -0.88 | -3.10 | 1.34 | 0.431 |  |  |  |
| ANB |  | -2.11 | -6.86 | 2.64 | 0.379 | 0.034 |  |  |
|  | Center M vs N | -1.30 | -3.18 | 0.57 | 0.171 |  |  |  |
|  | Milano born from 1999 onwards | 0.69 | -1.37 | 2.75 | 0.507 |  |  |  |
|  | Age | 0.10 | -0.12 | 0.31 | 0.373 |  |  |  |
|  | Sex M vs F | -1.72 | -3.19 | -0.26 | **0.022** |  |  |  |
| SNPg |  | 74.27 | 66.94 | 81.61 | 0.000 | 0.002 |  |  |
|  | Center M vs N | -0.07 | -2.97 | 2.83 | 0.963 |  |  |  |
|  | Milano born from 1999 onwards | 2.30 | -0.88 | 5.49 | 0.154 |  |  |  |
|  | Age | 0.12 | -0.22 | 0.45 | 0.485 |  |  |  |
|  | Sex M vs F | -0.15 | -2.42 | 2.12 | 0.894 |  |  |  |
| **Skeletal vertical** | |  |  |  |  |  |  |  |
| SN-NL |  | 8.10 | 1.82 | 14.38 | **0.012** | -0.002 |  |  |
|  | Center M vs N | 1.44 | -1.05 | 3.92 | 0.254 |  |  |  |
|  | Milan born from 1999 onwards | -1.92 | -4.65 | 0.80 | 0.165 |  |  |  |
|  | Age | 0.08 | -0.21 | 0.36 | 0.601 |  |  |  |
|  | Sex M vs F | -0.93 | -2.88 | 1.01 | 0.343 |  |  |  |
| SN-ML |  | 32.47 | 22.09 | 42.84 | 0.000 | 0.012 |  |  |
|  | Center M vs N | -2.56 | -6.66 | 1.54 | 0.219 |  |  |  |
|  | Milano born from 1999 onwards | -1.01 | -5.51 | 3.49 | 0.657 |  |  |  |
|  | Age | 0.13 | -0.34 | 0.60 | 0.584 |  |  |  |
|  | Sex M vs F | 0.32 | -2.89 | 3.52 | 0.845 |  |  |  |
| NL-ML |  | 24.39 | 11.37 | 37.40 | 0.000 | -0.004 |  |  |
|  | Center M vs N | -4.06 | -9.20 | 1.09 | 0.121 |  |  |  |
|  | Milano born from 1999 onwards | 2.00 | -3.65 | 7.64 | 0.484 |  |  |  |
|  | Age | 0.05 | -0.55 | 0.64 | 0.880 |  |  |  |
|  | Sex M vs F | 1.54 | -2.49 | 5.56 | 0.450 |  |  |  |
| RL-ML |  | 116.32 | 104.95 | 127.69 | 0.000 | 0.017 |  |  |
|  | Center M vs N | -1.49 | -5.98 | 3.01 | 0.512 |  |  |  |
|  | Milano born from 1999 onwards | 0.76 | -4.17 | 5.69 | 0.760 |  |  |  |
|  | Age | 0.48 | -0.04 | 0.99 | 0.070 |  |  |  |
|  | Sex M vs F | 2.44 | -1.07 | 5.96 | 0.171 |  |  |  |
| NaSBa |  | 124.03 | 116.24 | 131.81 | 0.000 | 0.053 |  |  |
|  | Center M vs N | 1.87 | -1.20 | 4.95 | 0.230 |  |  |  |
|  | Milano born from 1999 onwards | -3.41 | -6.79 | -0.04 | **0.048** |  |  |  |
|  | Age | 0.32 | -0.04 | 0.67 | 0.080 |  |  |  |
|  | Sex M vs F | 0.43 | -1.98 | 2.84 | 0.724 |  |  |  |
| **Dentoalveolar** |  |  |  |  |  |  |  |  |
| Ils-SN |  | 91.78 | 76.12 | 107.44 | 0.000 | 0.078 |  |  |
|  | Center M vs N | -2.13 | -8.32 | 4.06 | 0.496 |  |  |  |
|  | Milano born from 1999 onwards | 9.11 | 2.31 | 15.90 | **0.009** |  |  |  |
|  | Age | 0.52 | -0.19 | 1.23 | 0.152 |  |  |  |
|  | Sex M vs F | -2.29 | -7.13 | 2.56 | 0.351 |  |  |  |
| Ils-NL |  | 99.68 | 83.42 | 115.94 | 0.000 | 0.067 |  |  |
|  | Center M vs N | -0.53 | -6.96 | 5.90 | 0.870 |  |  |  |
|  | Milano born from 1999 onwards | 7.18 | 0.13 | 14.24 | **0.046** |  |  |  |
|  | Age | 0.60 | -0.14 | 1.33 | 0.112 |  |  |  |
|  | Sex M vs F | -3.00 | -8.03 | 2.03 | 0.239 |  |  |  |
| Interincisal |  | 137.95 | 117.05 | 158.86 | 0.000 | 0.010 |  |  |
|  | Center M vs N | 1.80 | -6.47 | 10.06 | 0.667 |  |  |  |
|  | Milano born from 1999 onwards | -6.55 | -15.62 | 2.52 | 0.155 |  |  |  |
|  | Age | -0.47 | -1.42 | 0.47 | 0.323 |  |  |  |
|  | Sex M vs F | 4.04 | -2.43 | 10.50 | 0.218 |  |  |  |
| ILI-ML |  | 97.75 | 85.34 | 110.17 | 0.000 | -0.011 |  |  |
|  | Center M vs N | 2.86 | -2.05 | 7.77 | 0.250 |  |  |  |
|  | Milano born from 1999 onwards | -1.53 | -6.92 | 3.85 | 0.573 |  |  |  |
|  | Age | -0.17 | -0.73 | 0.39 | 0.547 |  |  |  |
|  | Sex M vs F | -2.06 | -5.90 | 1.78 | 0.289 |  |  |  |
| Center M= Milano; Center N=Nijmegen; Gender M=Male; Gender F=Female | | | | |  |  |  |  |
